# Associations of land, cattle and food security with infant feeding practices among a rural population living in Manyara, Tanzania

**DOI:** 10.1186/s12889-018-5074-9

**Published:** 2018-01-19

**Authors:** Bailey Hanselman, Ramya Ambikapathi, Estomih Mduma, Erling Svensen, Laura E. Caulfield, Crystal L. Patil

**Affiliations:** 1 Department of Women, Children and Family Health Science, University of Illinois at Chicago, College of Nursing, Chicago, IL 60612 USA; 2 Fogarty International Center, National Institutes of Health, Bethesda, MD USA; 3 Department of Global Health and Population, Harvard T.H. Chan School of Public Health, Boston, MA USA; 4 Haydom Global Health Research Centre, Haydom, Tanzania; 5 Haukeland University Hospital, Bergen, Norway; 6 Department of International Health, The Johns Hopkins Bloomberg School of Public Health & Fogarty International Center, National Institutes of Health, Bethesda, MD USA

**Keywords:** Breastfeeding, Complementary feeding, Infant feeding, Nutrition, Land and livestock ownership, Food insecurity, Low-income countries, MAL-ED, Tanzania, Sub-Saharan Africa

## Abstract

**Background:**

Livelihoods strategies and food security experiences can positively and negatively affect infant and young child feeding (IYCF) practices. This study contributes to this literature by exploring how variation in household economics among rural farmers in Tanzania relates to IYCF patterns over the first 8 months of an infant’s life.

**Methods:**

These data were produced from a longitudinal study in which a cohort of mother-infant dyads was followed from birth to 24 months. In addition to baseline maternal, infant, and household characteristics, mothers were queried twice weekly and monthly about infant feeding practices and diet. Weekly and monthly datasets were merged and analyzed to assess infant feeding patterns through the first 8 months. Standard statistical methods including survival and logistic regression analyses were used.

**Results:**

Aside from breastfeeding initiation, all other IYCF practices were suboptimal in this cohort. Land and cattle ownership were associated with the early introduction of non-breastmilk food items. Food insecurity also played a role in patterning and inadequate complementary feeding was commonplace.

**Conclusions:**

Health promotion programs are needed to delay the introduction of animal milks and grain-based porridge, and to achieve a minimum acceptable diet after 6 months of age among smallholder farmers in rural Tanzania. Results highlight that livelihoods-based health promotion interventions, built from a flexible and integrated design, may be an important strategy to address community-level variation in infant feeding practices and promote optimal IYCF practices.

## Background

The strong relationship of optimal infant and young child feeding (IYCF) practices to growth and development and reduced risk for child morbidity and mortality is well-established [1–5]. In 2015, sub-Saharan Africa accounted for the most under-five deaths (2.9 million) [6, 7]. Tanzania is one of ten countries in the region accounting for 60% of all global under-five deaths [7]. In Tanzania more than a third of children under 24 months of age are stunted and a large proportion suffer from micronutrient deficiencies (iron, 42% and vitamin A deficiency, 33%) and anemia (73%) [6]. Given that malnutrition contributes to 45% of all under-five deaths, [8] improving IYCF practices remains an important global health priority [9, 10].

In Dar es Salaam, Tanzania’s urban center, the proportion of infants fed according to IYCF guidelines increases as mother’s education, wealth, and exposure to health messaging increases [6]. While such factors may be relevant to rural smallholder farmers, the relationship of these factors to farming livelihoods needs further clarification. Policy and intervention efforts aimed at improving IYCF practices need to account for relationships between IYCF practices and heterogeneity in livelihoods. We contribute to this discussion by assessing maternal and household socioeconomic indicators associated with IYCF patterns among a rural, smallholder farming community in the Manyara Region [11, 12]. These analyses provide a foundation from which to begin to disentangle the various ways that livelihoods strategies relate to infant feeding practices in places dependent on rain-fed and small-scale subsistence farming with high rates of stunting and micronutrient deficiencies [13].

## Methods

### Study design and subjects

This longitudinal, community-based prospective cohort study took place at the Haydom Tanzania (TZH) site located in the Manyara Region in north-central Tanzania. TZH was one of eight sites participating in the Etiology, Risk Factors and Interactions of Enteric Infections and Malnutrition and the Consequences for Child Health and Development (MAL-ED) study; a study designed to explore associations of etiology, risk factors, enteric infections, and dietary intake, to effects on child growth and cognitive development [14]. MAL-ED sites were selected based on epidemiological and geographical diversity, as well as, high rates of stunting and variable rates of diarrhea [14].

In brief, trained study personnel used a community survey to identify a sample of pregnant women. Most women were farmers with variable levels of market economy integration [13]. Inclusion criteria were: 1) healthy singleton newborn; 2) enrollment weight greater than 1500 g; 3) mother is greater than 16 years of age at time of study enrollment. At TZH, a total of 262 mother-infant pairs were recruited; 12 were lost to follow up before 170 days (8 dropped out, 3 passed away, and 1 was excluded due to > 25% data missing). A final sample of 250 mother-infant pairs was included in these analyses. All MAL-ED sites utilized a standardized protocol to ensure that data were comparable across sites [14, 15]. The TZH site and detailed information about poverty and malnutrition are described elsewhere [13]. Institutional Review Boards at each site and the collaborating institutions approved the protocol. Written informed consent was obtained for every participant.

### Data collection

Enrollment, biweekly, and monthly interview instruments were used to characterize infants’ key dietary exposures in months 1–9 [15, 16]. At the enrollment interview, trained personnel collected baseline demographic and household data including maternal age, parity, education, marital status, household characteristics, food security, [17] and early breastfeeding practices (first 24 hours after birth). Thereafter, household visits were made twice a week and once monthly to collect information on evolving infant feeding practices and to assess overall infant health (since the last contact, up to 7 days). The biweekly and monthly checklists allowed us to determine age of introduction and habitual consumption of non-breastmilk liquids, semi-solids, and solids.

Over the first 6 months, infants were visited a median of 51 times (interquartile range (IQR): 49, 53). At the 6 month follow up, water access and sanitation, eight assets, maternal education, and household income data were collected to construct a WAMI index to comprehensively assess household socioeconomic status [18]. Standard definitions were used to characterize breastfeeding status and practices [19]. The introduction of non-breastmilk liquid, solids, or semi-solids is defined as infant’s age in days at time of first reported introduction of non-breastmilk item, even if it was a single introduction and did not become a regular part of the infant’s diet. Though non-breastmilk nourishment can become habitual at any point after birth, the World Health Organization (WHO) differentiates habitual feeding from complementary feeding, in that complementary feeding is the recommended introduction of nutritious, safe food groups after 6 months of age, when breastmilk alone is no longer sufficient to meet the infant’s metabolic needs [1]. If non-breastmilk items were consumed on three visits in the last 10–12 days, the practice was categorized as habitual [20, 21]. We also evaluated non-breastmilk food introduction patterns and calculated the prevalence (in days) that various food items were present in the diet [21].

Modeled after questions on the Demographic and Health Surveys, a more extensive caretaker/mother monthly food frequency questionnaire was also used. From this data, we estimated the adequacy of complementary foods fed to infants between 6 and 8 months of age [15, 22]. Breastfeeding infants eating two or more meals per day met minimal standards for dietary frequency. If a breastfeeding infant ate foods from four or more food groups, their diet diversity was considered minimally diverse. A minimum acceptable diet (MAD) is a measure combining the dietary diversity (≥4 different food groups) and meal frequency (≥2 per day) standards [23]. The proportion of infants who consumed adequate iron-rich and vitamin A-rich foods were also calculated. Two measures of iron were used. The more restrictive measure included meats and organ meats, whereas the least restrictive measure included meats and organ meats plus fish, eggs, and leafy green vegetables.

### Statistical analyses

Descriptive analysis included examination of distribution of the variables, medians, and interquartile ranges. Duration of exclusive breastfeeding (EBF), predominant breastfeeding, and introduction of non-breastmilk foods were estimated using survival analysis. Personal prevalence of days with EBF, water, animal milk, and solids were constructed using the following calculation: first, proportion of total visits with EBF and non-breastmilk foods was estimated and then that total was multiplied by 180 days to yield personal prevalence. After bivariate analysis, a multivariate logistic regression model was constructed to assess factors associated with the early introduction (< 60 days) of non-breastmilk foods. The factors included were: gender, components of the WAMI index (household income, maternal education, improve water source/sanitation facility, assets), food security, land ownership, cattle ownership, maternal age, parity, type of first food given (water, animal milk, solids, other), and age at which first non-breastmilk food was introduced. When variables were collinear (e.g. parity and maternal age), a meaningful variable was kept for contextual relevance and interpretation. Normality of the outcome variables were tested prior to conducting the regression models. Data analyses for this study were conducted using STATA Version 13.1 (StataCorp LP, College Station, TX).

## Results

### Demographic and socio-economic characteristics

Infant, maternal, and household characteristics are presented in Table 1. The median infant age at enrollment was 7 days (IQR: 5–9). On average, infants weighed 3192 g at enrollment, just over half were male (51.0%), and nearly 11% were born to primigravid women, but most mothers had between 2 and 4 children. Mothers were on average 28 years of age and attended school for 5 years; most were married. A majority of households were food secure (68.9%), however more than a third experienced some level of food insecurity (31.3%) as measured by the HFIAS scale [17]. Over half of households owned 1–3 acres of land (52.0%) and more than two-thirds owned cattle (64.5%). The average per capita monthly income was calculated to be 35.8 USD.

**Table 1:** Select enrollment characteristics of mother-infant pairs (*n* = 250)

Characteristic	% or Mean (SD^a^)
Infant, median age at enrollment, days (25th, 75th percentiles)	7 (5,9)
Infant, enrollment weight, grams	3192 (441)
Infant, male	51
Parity, number of births	
1	10.8
2–4	47.8
> 4	41.4
Mother age, years	28.6 (6.6)
Mother education, years	5.0 (2.8)
Marital status, married	86.1
Any food insecurity [17]	31.3
Water and sanitation score [18]	1.9 (2.4)
Assets (# out of 8) [18]	2.0 (1.7)
Land ownership (%)	
None	5.2
1–3 acres	52.0
Owns 3+ acres of land	42.8
Cattle ownership, % yes	64.5
Per capita monthly income in USD	35.8^c^

^a^Standard Deviation

### Breastfeeding initiation

Breastfeeding practices occurring in the first 30 days of life for all eight MAL-ED sites are reported elsewhere [24]. At the THZ site, three-quarters (75.3%) of the infants met all three recommended breastfeeding initiation practices with 83.3% of infants put to breast within the first hour after birth. Nearly all of the infants were breastfed during their first day of life (98%), prelacteal feeding was rare (4.4%), and most newborns received colostrum (91.2%).

### Exclusive breastfeeding

**Fig. 1:**
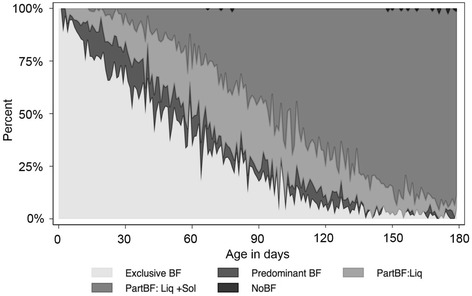
Prevalence plot of infant feeding patterns

A prevalence plot illustrates breastfeeding patterns over the first 180 days (Fig. 1). The median duration of exclusive breastfeeding was 38 days (IQR: 22, 66) and median duration of full breastfeeding was 50 days (IQR: 28, 74). Exclusive and full breastfeeding declined rapidly from 30 to 120 days. Exclusive breastfeeding declined from 62.0 to 1.6% and full breastfeeding declined from 72.4 to 4.0%. At 180 days, no infants were being exclusively breastfed. At 180 days, only 1.2% of infants were reported to have been fully breastfed on the day prior, 98.0% were partially breastfed, and only 0.8% had not received any breastmilk. Only four infants were reported as not breastfed during at least one visit in their first 180 days of life. Of these, two became fully weaned at 148 and 168 days. No infants were exclusively fed formula.

### Interruption of exclusive breastfeeding

Patterns associated with the introduction of non-breastmilk liquids and foods are in Table 2. Animal milk was the first and most commonly introduced non-breastmilk item introduced to 57.6% of the infants. The median age of introduction of animal milk was 51 days (IQR: 29, 80); only 18 infants (7.2%) did not receive animal milk during the first 6 months of life and all had received animal milk by the end of 8 months.

**Table 2:** Patterns in the introduction of non-breastmilk food items

	First Food (%)	Median introduction age, days (IQR)	Prevalence in the first 180 days, days (IQR)^a^
Animal milk	57.6	51 (29,80)	106 (65, 138)
Grains (maize porridge)	22.0	80 (55, 108)	88 (62, 114)
Animal milk + grains	8.0	50 (28, 74)	63 (29, 93)
Water	7.6	114 (72, 154)	7 (0,24)

^a^Proportion of visits with each food over total visits times 180

**Fig. 2:**
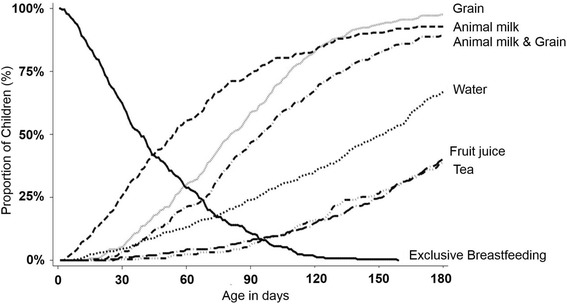
Introduction of non-breastmilk foods over the first 180 days

Grains, likely in the form of a semi-solid/solid maize porridge, represented the first food for 22.0% of infants. Nearly 8% of the infants received a combination of these two items (animal milk and grain) as their first food. Water (7.6%) or tea/coffee (1.6%) was the first food for the remainder of infants. Figure 2 illustrates patterns in the proportion of infants receiving these non-breastmilk food items across the first 180 days.

### Factors associated with the early introduction of non-breastmilk foods

Logistic regression modeling was used to assess factors associated with three infant feeding practices (Table 3). The first column represents factors related to the introduction of any non-breastmilk foods to the infant prior to 60 days. The second and third columns focus on the interruption of exclusive breastfeeding from the two most commonly introduced food items, animal milks and grains.

**Table 3:** Results of logistic regression models assessing relationships among socioeconomic variables and the introduction of non-breastmilk foods before 60 days

	Introduction of non-breastmilk foods < 60 days		
	Any food	Animal milk	Grains
Female	0.640	1.196	0.683
	[0.357,1.149]	[0.678,2.108]	[0.373,1.250]
First born	0.501	0.731	0.664
	[0.205,1.227]	[0.300,1.778]	[0.250,1.765]
Income	0.999	0.999	*0.982* ^*^
	[0.991,1.007]	[0.991,1.007]	*[0.966,0.998]*
Sanitation score	*1.141* ^*^	*1.106* ^*+*^	1.098
	*[1.014,1.284]*	*[0.986,1.241]*	[0.970,1.244]
Assets	0.955	0.913	*0.745* ^*^
	[0.799,1.142]	[0.766,1.090]	*[0.601,0.923]*
Own cattle	1.429	*1.932* ^*^	0.840
	[0.757,2.698]	*[1.047,3.566]*	[0.438,1.613]
Own > 3 acres of land	0.640	0.699	0.584
	[0.342,1.195]	[0.343,1.427]	[0.301,1.134]
Any food insecurity	0.636	*0.387* ^*^	0.611
	[0.342,1.182]	*[0.175,0.859]*	[0.311,1.200]
Own land + food insecure	a	*2.907* ^*+*^	a
		*[0.841,10.04]*	
Observations	242	224	236

Exponentiated coefficients; 95% confidence intervals in brackets

*p < 0.05, ^**+**^*p* < 0.10

^a^Interaction variables were not included because differences in marginal probabilities were not significant and did not improve the model fit

The only factor related to the early introduction of any non-breastmilk foods (first column) was a higher sanitation score; higher sanitation increased the likelihood of introduction of any non-breastmilk foods before 60 days by 14% (*p* = 0.029). A higher sanitation score also increased the likelihood of introduction of animal milk before 60 days by 10% (*p* = 0.086). Sanitation, however, was not related to the introduction of grains before 60 days. Having a higher income reduced the likelihood of introducing grains to infants before 60 days by 8%. Income had no relationship to the overall introduction of non-breastmilk foods or animal milks before 60 days. A factor that reduced the likelihood of introducing grains to infants before 60 days was household assets. Households with more assets were 25% (*p* = 0.007) less likely to introduce grains before 60 days.

**Fig. 3:**
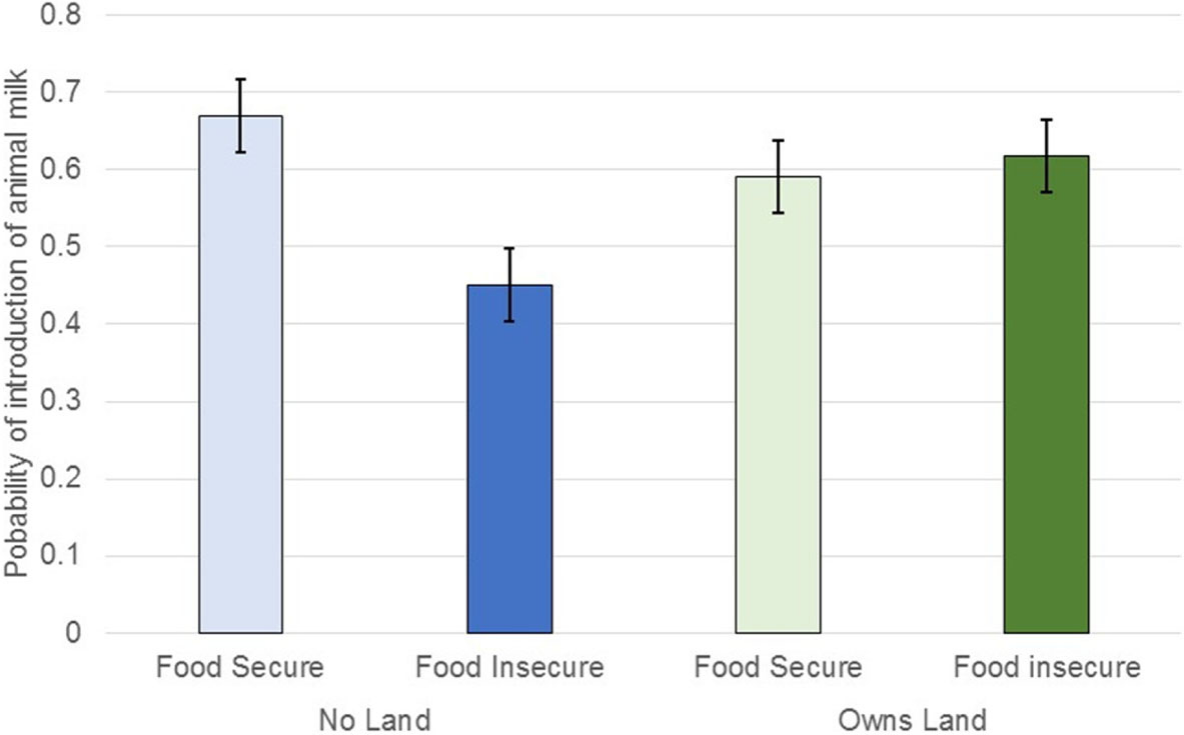
Marginal probabilities of owning land and food insecurity on introduction of animal milk to the infant before 60 days (see Table 3)

Two-way interaction terms were tested between food insecurity, land ownership (> 3 acres), and cattle ownership (Table 3). Of these interactions, we identified that there is a significant interaction between land ownership and food insecurity (Fig. 3). Food insecurity alone reduced the likelihood of early introduction of animal milk by 62% (*p* = 0.020). However, among the group that is both food insecure and owns land, there is a 66% (*p* = 0.092) further reduction of the likelihood of early animal milk introduction. There were no significant differences of land ownership on the food secure group. Furthermore, we did not find any significant impact with interaction terms in the other two models.

To begin to disentangle some of these results, we speculated that the income variable may have absorbed the effect of land ownership on the early introduction of foods. To test this hypothesis, we excluded the income variable and retained the land ownership variable in a model for early introduction of grains. This model showed a statistically significant relationship between land ownership and grain introduction, where land ownership reduced the likelihood of early introduction by 53% (*p* = 0.056). In addition, we also explored the influence of birth month on the outcome to evaluate the impact of crop production or seasonal availability of dairy on introduction of foods. There were no associations between birth month on introduction of animal milk or non-breastmilk foods. There was marginal association in the bivariate model for introduction of grain. So another multivariate model was created, where we found that infants born between June to December had higher likelihoods for introduction of grains (results not shown).

### Complementary feeding practices

**Fig. 4:**
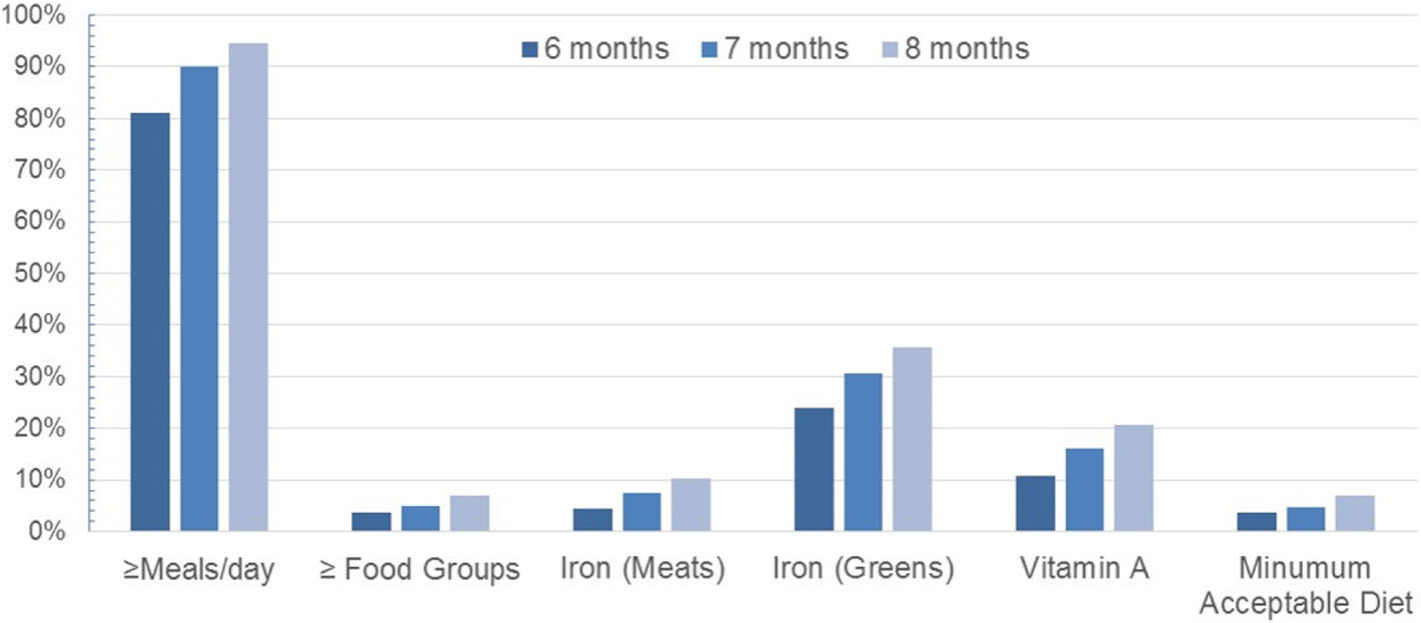
Percentage of infants meeting the WHO’s complementary feeding core indicators for quality at 6, 7 and 8 months of age estimated from the monthly questionnaire

For infants between 6 and 8 months of age, the quality and adequacy of the complementary feeding diet was estimated from food frequency data collected on the monthly questionnaire. At 8 months of age, all infants were habitually eating solid or semi-solid foods in addition to breastmilk and/or animal milk (Fig. 4). Dietary diversity in months 6, 7 and 8 was low. No more than 7.1% of infants received four or more different food groups on the day prior to the monthly interview. Meal frequency was higher than dietary diversity; 81.0% of 6-month-old infants, 90.1% of 7 month-olds, and 94.6% of 8-month olds ate two or more meals the day prior to the interview. Though meal frequency was high, the proportion of infants with minimally acceptable diets across months 6–8 ranged from 3.7 to 7.1%. Iron-rich foods (greens), the least restrictive definition, were consumed by 23.9–35.8% of the infants between 6 and 8 months of age. Fewer infants (10.7–20.8%) consumed vitamin-A rich containing foods.

## Discussion

Associations between optimal infant feeding and health are well-recognized and Tanzania has set regional and national targets to increase the prevalence of exclusive breastfeeding and improve infant and young child feeding practices [25]. Rates of breastfeeding initiation within 1 hour of birth were higher in the TZH cohort (83.3%) compared to national rates (51.0%) and those reported for the Manyara Region (75.0%) [25]. Also, fewer TZH infants received a prelacteal feed (4.4%) compared to data reported from the Manyara Region (11.6%).

Aside from breastfeeding initiation outcomes, all other IYCF practices were suboptimal for the TZH cohort. Nationally, it is reported that 59.0% of infants are exclusively breastfed. However, none in the TZH cohort met this recommendation; the median duration of EBF was just 38 days. The infant with longest reported duration of EBF was 158 days. By 90 days, less than a quarter of the TZH infants were exclusively breastfed. Our findings indicate that there is an early and steady decline in EBF over the first 8 months of life [24].

Participants in this study are primarily smallholder farmers, so we investigated factors related to farming livelihoods and described how cattle, land ownership, and food security relate to infant feeding practices over the first 8 months of life. Our results substantiate those reported from a Kenyan study which showed that dairy producing households had a 12-fold increased risk for early animal milk introduction compared to those in households without cattle [26]. Similarly, our data also show that cattle ownership adversely affects infant feeding practices. TZH farmers owning cattle were more likely to introduce animal milk before 60 days. This pattern is even earlier than that reported among a neighboring pastoralist ethnic group whose livelihoods center on cattle and milk [27]. Cattle can be raised and sold to increase household income; however,  in this context cattle are often used as form of savings to be sold in emergency situations. The role of cattle is important because owning indicates that a household has some financial flexibility and stability. In support of this observation, TZH infants from food insecure households were less likely to receive cow milk early. This relationship was exacerbated among participants who did not own land and were experiencing food insecurity.

In this region, maize is the dietary staple; meals are not considered complete without the inclusion of maize-based stiff porridge. Maize also plays an important role in household economics and represents a critical component of market integration. In addition to other crops (e.g., sunflowers and legumes), maize provides a critical source of income for purchases (e.g., soap, roofing materials, cell phones, etc.) and expenses, such as school fees and healthcare. Households strategize about the selling of their excess maize stores; typically, households will sell maize during the rainy season (January–March) and before the next harvest when the prices are highest. If maize stores are sold and the sale is followed by poor rains or a poor harvest, this strategy has implications for food insecurity and future household economic stability.

Unlike cattle ownership and its link to the early introduction of milk, land ownership was not associated with the early introduction of grains. But, there was an inverse relationship between higher income and the early introduction of grains. From this we posit that income derived from land ownership is not used for buying foods for infants, or that crops cultivated from the land are sold rather than consumed. This may explain why there is a lower likelihood of grain introduction. Land ownership did not have an independent effect on the early introduction of animal milk even in households experiencing food insecurity.

Decision-making about how crops and animals are utilized within the larger household economy are also unclear at this time, but may be linked to gender roles and expectations. From field observations, we noted that chickens, which freely roam around the household compound, are cared for by women and children. Women are often able to make independent decisions about when to cook or sell chickens and/or eggs. Interestingly, however, close contact with chickens appears to increase exposure to *Campylobacter* and can lead to poorer health outcomes for infants and children [28, 29]. While some households describe having shared decision-making strategies, it is far more common for men to make decisions about crops and the larger animals. Additional information about household planning and the role of other livestock (e.g., pigs, chicken, goats and sheep) in smallholder farming livelihoods needs further investigation.

With the Millennium Development Goals and their associated interventions, tremendous progress was made to improve infant nutritional health. At TZH, it appears that national programming implemented locally through antenatal care and maternity services has positively affected breastfeeding initiation. However, if resources and health promotion remain focused at the national level, progress will likely stall. Both iron- and vitamin A rich foods are available in this community, but what is unclear is why so few in the TZH cohort include these foods in their infants’ diets. More work is needed to address poor exclusive breastfeeding rates, low dietary diversity, and in designing locally appropriate interventions.

## Conclusions

Community-level variation related to smallholder farming livelihoods clearly plays a role in how infants are fed. We identified that cattle ownership increases the early introduction of animal milks. We also showed that owning more land appears to be a risk factor for the early introduction of non-breastmilk food items, but we cannot draw a causative conclusion from the data collected for this study. Like others, this research shows that there is a relationship between food security and infant feeding practices [30, 31]. Interventions (e.g. agricultural and nutritional education) can be designed to consider community-level variation to account for differences in the everyday lives of community members, such as decisions about feeding children in relation to livelihoods strategies, agriculture, and livestock production. Based on these findings, additional research is needed to delineate the multiple pathways by which smallholder farming, livelihoods strategies, land and animal ownership, and infant feeding practices interact [32]. To be most effective, programs and policies that target homestead gardening as a nutrition-sensitive approach to improving IYCF will need to creatively examine household decision-making such as, the flows of money from selling crops and animals, and how this economy relates to gender, empowerment, infant feeding practices, and short- and long-term nutritional outcomes [32–35].

## Abbreviations

EBF: Exclusive breastfeeding
IQR: Interquartile range
IYCF: Infant and young child feeding
MAD: Minimum acceptable diet
MAL-ED: The Etiology, Risk Factors and Interactions of Enteric Infections and Malnutrition and the Consequences for Child Health and Development
TZH: Haydom, Tanzania (study site)
WAMI index: Water and sanitation, assets, maternal education, and household income
WHO: World Health Organization

## References

[1] WHO/UNICEF. Global Strategy for Infant and Young Child Feeding. Geneva: World Health Organization; 2003.

[2] Jones D, Weiss S, Chitalu N. HIV Prevention in Resource Limited Settings: A Case Study of Challenges and Opportunities for Implementation. Int J Behav Med. 2014;22(3):384-92. Available from: http://www.ncbi.nlm.nih.gov/pubmed/24604206. 10.1007/s12529-014-9397-3 PMC4721251 24604206

[3] Black RE, Allen LH, Bhutta ZA, Caulfield LE, de Onis M, Ezzati M, et al. Maternal and child undernutrition: global and regional exposures and health consequences. Lancet. 2008;371:243–60. Available from: http://www.sciencedirect.com/science/article/pii/S0140673607616900. 10.1016/S0140-6736(07)61690-0 18207566

[4] Caulfield LE, de Onis M, Blassner M, Black RE. Undernutrition as an underlying cause of child deaths associated with diarrhea, pneumonia, malaria, and measles. Am J Clin Nutr. 2004;80:193–8. Available from: http://www.ajcn.org/content/80/1/193.abstract. 10.1093/ajcn/80.1.193 15213048

[5] WHO. Complementary feeding of young children in developing countries: A review of current scientific knowledge. Geneva: WHO; 1998. Available from: http://www.who.int/nutrition/publications/infantfeeding/WHO_NUT_98.1/en/.

[6] Vitta BS, Benjamin M, Pries AM, Champeny M, Zehner E, Huffman SL. Infant and young child feeding practices among children under 2 years of age and maternal exposure to infant and young child feeding messages and promotions in Dar es salaam. Tanzania Matern Child Nutr. 2016;12:77–90. Available from: https://www.ncbi.nlm.nih.gov/pubmed/27061958. 10.1111/mcn.12292 PMC5071773 27061958

[7] Liu L, Oza S, Hogan D, Chu Y, Perin JZ, Lawn JE, et al. Global, regional, and national causes of child mortality in 2000–13, with projections to inform post-2015 priorities: an updated systematic analysis. Lancet. 2016;385:430-40. Available from: http://www.sciencedirect.com/science/article/pii/S0140673614616986. 10.1016/S0140-6736(14)61698-6 25280870

[8] Black RE, Victora CG, Walker SP, Bhutta ZA, Christian P, de Onis M, et al. Maternal and child undernutrition and overweight in low-income and middle-income countries. Lancet. 2013;382:427–51. Available from: https://www.ncbi.nlm.nih.gov/pubmed/23746772. 10.1016/S0140-6736(13)60937-X 23746772

[9] IFPRI. Global Nutrition Report 2016: From Promise to Impact: Ending Malnutrition by 2030. Washington, D.C.: International Food Policy Research Institute. 2016. Available from: http://dx.doi.org/10.2499/9780896295841

[10] FAO. The State of Food Insecurity in the World. Rome: FAO; 2015. Available from: http://www.fao.org/3/a-i4646e.pdf.

[11] Koppmair S, Kassie M, Qaim M. Farm production, market access and dietary diversity in Malawi. Public Health Nutr. 2017;20:325–35. Available from: https://www.ncbi.nlm.nih.gov/pmc/articles/PMC5244442/. 10.1017/S1368980016002135 PMC5244442 27609557

[12] Frelat R, Lopez-Ridaura S, Giller KE, Herrero M, Douxchamps S, Andersson Djurfeldt A, et al. Drivers of household food availability in sub-Saharan Africa based on big data from small farms. Proc Natl Acad Sci. 2016;113:458–63. Available from: http://www.ncbi.nlm.nih.gov/pubmed/26712016. 10.1073/pnas.1518384112 PMC4720294 26712016

[13] Mduma ER, Gratz J, Patil C, Matson K, Dakay M, Liu S, et al. The Etiology, Risk Factors, and Interactions of Enteric Infections and Malnutrition and the Consequences for Child Health and Development Study (MAL-ED): Description of the Tanzanian Site. Clin Infect Dis. 2014;59:S325–30. Available from: http://cid.oxfordjournals.org/content/59/suppl_4/S325.abstract. 10.1093/cid/ciu439 25305305

[14] The MAL-ED Network. The MAL-ED study: a multinational and multidisciplinary approach to understand the relationship between enteric pathogens, malnutrition, gut physiology, physical growth, cognitive development, and immune responses in infants and children up to 2 years of. Clin Infect Dis. 2014;59:S193–S206. Available from: http://cid.oxfordjournals.org/content/59/suppl_4/S193.abstract. 10.1093/cid/ciu653 25305287

[15] Caulfield LE, Bose A, Chandyo RK, Nesamvuni C, de Moraes ML, Turab A, et al. Infant Feeding Practices, Dietary Adequacy, and Micronutrient Status Measures in the MAL-ED Study. Clin Infect Dis. 2014; 59:S248–54. Available from: http://cid.oxfordjournals.org/content/59/suppl_4/S248.abstract. 10.1093/cid/ciu421 PMC4204612 25305294

[16] Gibson R. Priniciples of Nutritional Assessment. Oxford: Oxford University Press; 2005.

[17] Coates J, Swindale A, Bilinsky P. Household Food Insecurity Access Scale (HFIAS) for Measurement of Food Access: Indicator Guide (v. 3). Washington D.C.: Food and Nutrition Technical Assistance Project (FANTA); 2007. Available from: http://www.fantaproject.org/sites/default/files/resources/HFIAS_ENG_v3_Aug07.pdf.

[18] Psaki SR, Seidman JC, Miller M, Gottlieb M, Bhutta ZA, Ahmed T, et al. Measuring socioeconomic status in multicountry studies: results from the eight-country MAL-ED study. Popul Health Metr. 2014;12:1–11. Available from: https://www.ncbi.nlm.nih.gov/pubmed/24656134. 10.1186/1478-7954-12-8 PMC4234146 24656134

[19] Labbok MH, Krasovec K. Toward consistency in breastfeeding definitions. Studies in Family Planning. 1990;21:226–30. http://dx.doi.org/10.2307/1966617. 2219227

[20] Lee G, Paredes Olortegui M, Rengifo Pinedo S, Ambikapathi R, Peñataro Yori P, Kosek M, et al. Infant feeding practices in the Peruvian Amazon: implications for programs to improve feeding. Rev. Panam. Salud Pública. Organización Panamericana de la Salud; 36:150–7. Available from: https://www.ncbi.nlm.nih.gov/pubmed/25418764. 25418764

[21] Ambikapahthi R, Kosek M, Olortegui M, Caulfield L. High resolution longitudinal analysis to evaluate the timing, duration and dynamics of exclusive breastfeeding in the Peruvian Amazon. Federation of American Societies for Experimental Biology. 2014;28:119.3. Available from: http://www.fasebj.org/doi/10.1096/fasebj.28.1_supplement.119.3.

[22] Demographic and Health Surveys. Survey methodology - survey process. 2010. Available from: http://preview.dhsprogram.com/What-We-Do/Survey-Types/DHS-Questionnaires.cfm.

[23] WHO. Indicators for Assessing Infant and Young Child Feeding Practices. Part 2: Measurement. Geneva: World Health Organization; 2010. Available from: http://www.who.int/nutrition/publications/infantfeeding/9789241599290/en/.

[24] Patil CL, Turab A, Ambikapathi R, Nesamvuni C, Chandyo RK, Bose A, et al. Early interruption of exclusive breastfeeding: results from the eight-country MAL-ED study. J. Health. Popul. Nutr. BioMed Central. 2015;34:10. Available from: http://jhpn.biomedcentral.com/articles/10.1186/s41043-015-0004-2. 10.1186/s41043-015-0004-2 PMC5025973 26825923

[25] MoHCDGEC. Tanzania Demographic and Health Survey and Malaria Indicator Survey (TDHS-MIS) 2015–16. Dar es Salaam, Tanzania, and Rockville, Maryland, USA: Ministry of Health, Community Development, Gender, Elderly and Children (MoHCDGEC), [Tanzania Mainland], Ministry of Health (MoH) [Zanzibar], National Bureau of Statistics (NBS), Office of the Chief Government Statistician (OCGS), and ICF International; 2016. Available from: https://dhsprogram.com/pubs/pdf/FR321/FR321.pdf.

[26] Wyatt AJ, Yount KM, Null C, Ramakrishnan U, Webb Girard A. Dairy intensification, mothers and children: an exploration of infant and young child feeding practices among rural dairy farmers in Kenya. Matern. Child Nutr. 2015;11:88–103. Available from: http://doi.wiley.com/10.1111/mcn.12074. 10.1111/mcn.12074 PMC6860198 23941354

[27] Sellen D (1998). Infant and young child feeding practices among African pastoralists: the Datoga of Tanzania. J Biosoc Sci.

[28] George CM, Oldja L, Biswas SK, Perin J, Lee GO, Ahmed S, et al. Fecal Markers of Environmental Enteropathy are Associated with Animal Exposure and Caregiver Hygiene in Bangladesh. Am J Trop Med Hyg. 2015;93:269–75. Available from: https://www.ncbi.nlm.nih.gov/pubmed/26055734. 10.4269/ajtmh.14-0694 PMC4530746 26055734

[29] Kaur M, Graham JP, Eisenberg JNS. Livestock Ownership among Rural Households and Child Morbidity and Mortality: An Analysis of Demographic Health Survey Data from 30 Sub-Saharan African Countries (2005–2015). Am J Trop Med Hyg. 2017;16–0664. Available from: http://www.ncbi.nlm.nih.gov/pubmed/28044044. 10.4269/ajtmh.16-0664 PMC5361555 28044044

[30] Webb-Girard A, Cherobon A, Mbugua S, Kamau-Mbuthia E, Amin A, Sellen DW. Food insecurity is associated with attitudes towards exclusive breastfeeding among women in urban Kenya. Matern. Child Nutr. 2012;8:199–214. Available from: http://doi.wiley.com/10.1111/j.1740-8709.2010.00272.x. 10.1111/j.1740-8709.2010.00272.x PMC6860665 20874844

[31] Marquis GS, Lartey A, Perez-Escamilla R, Mazur RE, Brakohiapa L, Birks KA, et al. Factors are not the same for risk of stopping exclusive breast-feeding and introducing different types of liquids and solids in HIV-affected communities in Ghana. Br. J. Nutr. 2016;116:115–25. Available from: http://www.journals.cambridge.org/abstract_S0007114516001707. 10.1017/S0007114516001707 27149980

[32] Ruel MT, Alderman H (2013). Nutrition-sensitive interventions and programmes: how can they help to accelerate progress in improving maternal and child nutrition?. Lancet.

[33] Na M, Jennings L, Talegawkar SA, Ahmed S, Black RE, Victora CG, et al. Association between women’s empowerment and infant and child feeding practices in sub-Saharan Africa: an analysis of Demographic and Health Surveys. Public Health Nutr. [Internet]. 2010; 5:e11190. Available from: http://www.journals.cambridge.org/abstract_S1368980015002621. 10.1017/S1368980015002621 PMC10271619 26347195

[34] Bezner Kerr R, Shumba L, Dakishoni L, Lupafya E, Berti P, Classen L, et al. Participatory, Agroecological and gender-sensitive approaches to improved nutrition: a case study in Malawi. 2013. Available from: http://www.fao.org/3/a-as559e.pdf.

[35] Micere Njuki J, Wyatt A, Baltenweck I, Yount K, Null C, Ramakrishnan U, et al. An Exploratory study of Dairying Intensification, Women’s Decision Making, and Time Use and Implications for Child Nutrition in Kenya. Eur. J. Dev. Res. 2016;28:722–40. Available from: http://link.springer.com/10.1057/ejdr.2015.22.

